# The applicability of a cueing paradigm to study individual differences in the spotlight of attention

**DOI:** 10.3758/s13414-025-03136-0

**Published:** 2025-07-29

**Authors:** Beleke de Zwart, Dirk van Moorselaar, Antonia F. Ten Brink, Stefan Van der Stigchel

**Affiliations:** 1 Experimental Psychology, Helmholtz Institute, Utrecht University, Heidelberglaan 1, 3584 CS Utrecht, The Netherlands; 2 Center of Excellence for Rehabilitation Medicine, UMC Utrecht Brain Center, University Medical Center Utrecht, and De Hoogstraat Rehabilitation, Utrecht, the Netherlands

**Keywords:** Attention, Cueing, Individual differences

## Abstract

Our spotlight of attention allows selecting and filtering relevant information from the world around us, and thereby influences how we perceive the world. However, it remains poorly understood whether there are, next to state influences, also stable differences in the sharpness of the spotlight between individuals: trait differences. Therefore, our aim was to investigate the validity of a cueing paradigm to assess group *and* individual differences. In an online experiment, we presented a cue to covertly direct attention to one side of the screen. This was followed by a target (gapped circle) appearing at varying distances from the cue, in either the valid or the invalid hemifield. The accuracy of reporting the gap direction was used to map the size of the attentional spotlight. First, results indicated that our task elicited the standard exogeneous cueing effects. Then, we fitted linear slopes to index the spatial gradient of the spotlight and demonstrated performance decline as a function of increasing target distance, showing that attention modulates the sharpness of the spotlight. Test-retest analyses revealed that while the patterns observed at the group level are robust, performance on the individual level was not reliably stable over a 2-week period, limiting the validity of the cueing paradigm for individual differences research. Whereas not suitable to detect variance in the homogeneous neurotypical population, we discuss potential use of the task for future research in clinical populations where altered attentional functioning are hallmarks in the clinical diagnosis (e.g., autism spectrum conditions), and in reassessing previously reported group level differences.

**Supplementary Information:**

The online version contains supplementary material available at 10.3758/s13414-025-03136-0.

## Introduction

Our visual environment presents far more information than our cognitive systems can process simultaneously, necessitating mechanisms to focus on relevant stimuli and filter out distractions. Attention plays this crucial role, shaping our perception and determining our experience of the world. A common way to characterize the distribution of attentional resources across space is through the “zoom lens” or “spotlight” metaphor (Cave & Bichot, 1999; Eriksen & James, 1986). These models posit two key features of attention:

1. A gradient of processing quality that declines with distance from the central focus (Beck & Ambler, 1973; Downing, 1988; Eriksen & James, 1986), and
2. An inverse relationship between processing quality and the size of the attended region (Castiello & Umiltà, 1990; LaBerge, 1983). This model explains how narrowing attention leads to enhanced detail discrimination, while a broader focus allows perception of larger areas, demonstrating the flexibility of attentional processes in adapting to various cognitive demands.

The size of the attentional spotlight is known to be modulated by situational demands. For example, when walking through a busy city, you broaden your attentional spotlight to encompass the global environment, ignoring irrelevant details like the book another pedestrian is holding. However, when you are in a restaurant, deciding what you want to eat, the spotlight narrows to focus on the local information, enabling you to read the menu. This ability to adjust the size of the attentional spotlight has widely been documented. For instance, Kolnes et al. (2024) showed that attentional breadth can be manipulated using auditory cues, while Belopolsky and Theeuwes (2010) found that when participants successfully focused narrowly in a singleton task, distractors outside the attentional spotlight were prevented from attentional capture.

Alongside the empirical evidence showing that attentional breadth is a flexible mechanism which can be adjusted depending on task demands, research has also characterized various spatial configurations of attention. There is substantial evidence supporting different attentional topologies including a Mexican hat profile with a central peak surrounded by inhibitory regions (Müller et al., 2005), an elliptical configuration that extends along specific axes (Tse et al., 2003), and annular or ring-shaped distributions (e.g., Egly & Homa, 1984), with the different topologies influenced by factors such as the task and dependent measure. Despite extensive research on the state-driven modulation of attentional breadth, however, it remains unclear whether stable individual differences – “trait differences” – also exist. As selection and prioritization of information through attention is also influenced by factors such as experience and expertise, this might hold for the default differences in the spotlight size as well. Some individuals may have a natural preference for a broader, global attentional focus, whereas others may be more inclined toward detailed, fine-grained processing.

Previous studies have investigated trait differences in attentional processing by comparing various groups, including different cultures, religions, and clinical populations (e.g., Colzato et al., 2010; McKone et al., 2010; Ten Brink et al., 2024). These studies typically employ tasks such as NAVON, Kimchi Palmer, or other hierarchical shape tasks to index local versus global processing (Fink et al., 1997; Kimchi & Palmer, 1982; Navon, 1977). For instance, NAVON stimuli consist of large letters composed of smaller letters, requiring participants to attend either the global (large) or the local (small) perceptual level (Navon, 1977). Generally, these studies have observed a global precedence effect, indicating faster identification of global features compared to local characteristics. Another paradigm used to compare trait differences in visual performance is the Useful Field of View task (e.g., Boduroglu & Shah, 2017; Coeckelbergh et al., 2004; Sekuler & Bennett, 2000). The Useful Field of View task was designed to assess the functional range of peripheral vision under conditions of varying cognitive load (Aust & Edwards, 2016; Wood & Owsley, 2014; Woutersen et al., 2017). Critically, however, these tasks typically measure multiple cognitive processes simultaneously, making it challenging to isolate the specific construct of attentional spotlight size (e.g., Busch et al., 2024; Dale & Arnell, 2013; Medaglia et al., 2017; Woutersen et al., 2017).

Given these limitations, the current work aimed to investigate whether a cueing paradigm can serve as a valid measure to assess the *sharpness of the gradient of the attentional spotlight*. This approach builds on the work by Robertson et al. (2013), who adapted Posner's (1980) cueing paradigm. Specifically, Robertson et al. (2013) used exogenous cues at varying distances from the target (i.e., a gapped circle) to assess the sharpness of the spotlight borders. They compared performance between individuals with and without a diagnosis of Autism Spectrum Disorder (ASD) and found that participants with autism exhibited a sharper gradient of attention, characterized by a greater fall-off in the ability to detect the direction of the target gap with increasing cue-target distance. This finding aligns with robust evidence in the literature reporting less interference from crowding and more efficient detail perception in visual scenes for individuals with ASD (Baldassi et al., 2009; Chung & Son, 2020). Although this cueing task is a promising tool to study differences in the size of the attentional spotlight, to date it has only been used to compare groups, leaving it unclear whether it can also reliably track stable individual differences (Hedge et al., 2018).

Traditionally, within common experimentalist frameworks, individual differences were often considered noise or unexplained error variance. However, this approach provides an incomplete picture of cognitive functioning across a population and overlooks valuable insights into the diversity of our society. A growing body of literature now emphasizes that paradigms used to investigate between-group differences cannot be blindly applied to study within-group differences (Goodhew & Edwards, 2019; Hedge et al., 2018). This shift in perspective highlights the importance of developing and validating measures that can effectively capture individual variations in attentional processes, such as the sharpness of the attentional spotlight gradient.

The present study aimed to explore the potential of using a cueing paradigm to assess both group-level performance and individual variations in attentional processes. Building on the well-established cueing effects in the literature (Posner, 1980; but see Intriligator & Cavanagh (2001) for limitations of this approach in measuring fine-grained attentional resolution), and the paradigm’s ability to provide insights into atypical cognitive functioning (Robertson et al., 2013), we investigated its applicability for individual differences research through high-powered online studies. Specifically, we assessed whether the task provided a stable index of the spotlight gradient through test-retest analysis. *If* these spotlight differences were stable within individuals over time, we could conceptualize the attentional gradient more as a continuous measure.

In addition to theoretical advancement, understanding individual variation in attentional breadth has wide-ranging implications for society. For example, it could improve our understanding of populations where altered attentional functioning is a clinical hallmark, such as ASD or Attention-Deficit (Hyperactivity) Disorder (ADHD). This can improve the diagnostic process by characterizing the nature of attentional differences rather than solely indicating their presence. In turn, this can benefit person-centered interventions. This highlights the importance of finding reliable measures to assess these individual differences, and we therefore hope to establish our paradigm as a reliable index of attentional spotlight characteristics.

## Methods

### Participants

Participants were recruited via the online platform Prolific (http://www.prolific.co) and provided digital informed consent. The experiment complied with all ethical guidelines set out in the Declaration of Helsinki, and was approved by the Ethics Committee of the Faculty of Social and Behavioral Sciences of Utrecht University. The monetary reward for successful completion of the task was (the equivalent of) 7.65 GBP. As there were no prior comparable studies run online, we based the sample size on a reliability study of the Posner cueing task of Hedge et al. (2018). Sixty participants (aged 18–35 years; located in Europe and fluent in English) completed the first experimental session, of whom 56 also completed the second session (M = 14.3 days in between sessions, SD = 1.02 day). Two participants were excluded as they indicated having a formal diagnosis of ASD and one participant who performed more than three blocks due to a fault in the system was removed. This resulted in 57 participants in Session 1 (21 female, 36 male, *M =* 25.81 years old, *SD =* 4.73 years) and 53 in Session 2.

### Apparatus and stimuli

The experiment was programmed using the JavaScript libraries jsPsych (7.0; http://www.jsPsych.org/v7) and was hosted online via the web service Gorilla (www.gorilla.sc). To control for the inevitable variation of monitor sizes, we implemented a standard calibration procedure at the beginning of the task (Yung et al., 2015). Participants were asked to resize a rectangle on the screen to match a card with a standard size (e.g., credit card, commonly 8.56 cm wide; participants were excluded if the rescaling factor fell outside the acceptable range of < 0.8 or > 1.3.). Participants were instructed to position themselves right in front of the screen, sit straight in their chair, and be one arm’s length distance away. We asked participants explicitly to perform Session 2 in the exact same setting as Session 1.

### Attention task

**Fig. 1:**
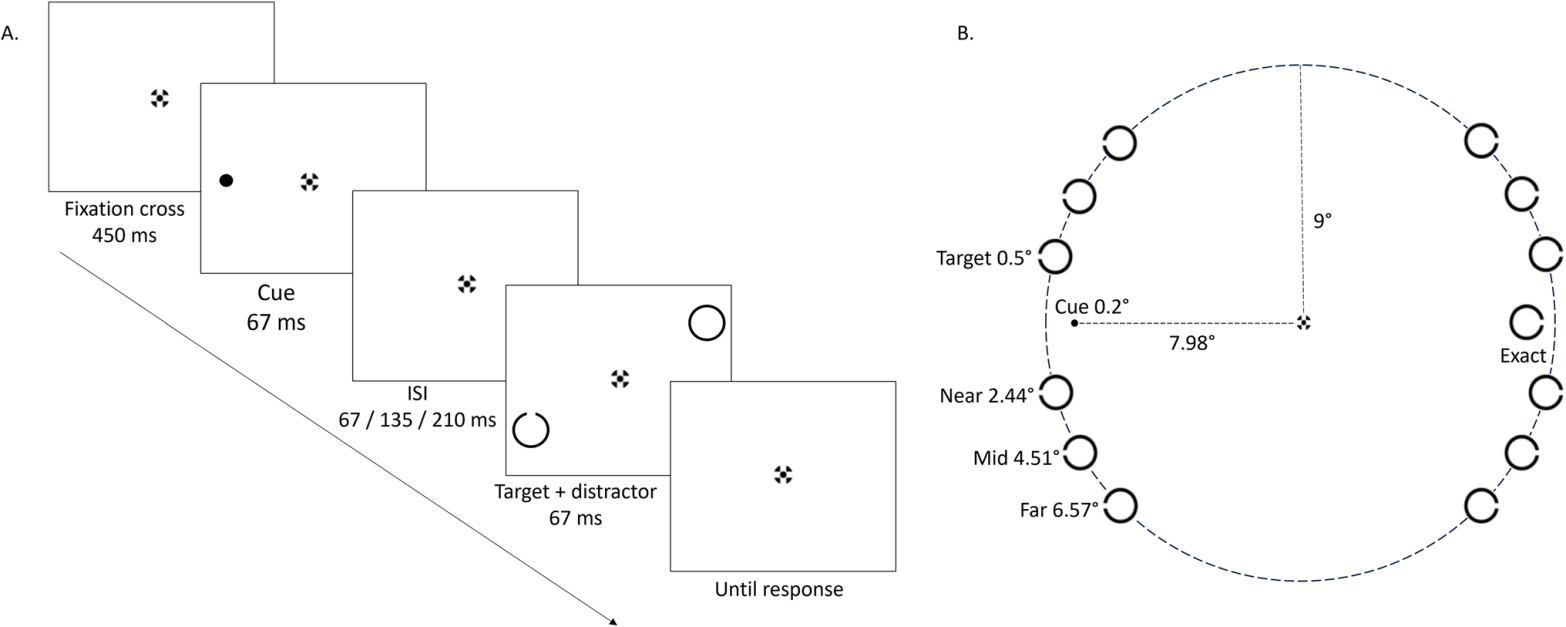
(**A**) Example of an experimental trial in a valid condition (not shown to scale). Each trial starts with a fixation point, followed by a cue. After a variable interstimulus interval (67, 135, or 210 ms), the target appears (here at target distance *mid*) simultaneously with a distractor (180° opposite of the target). Participants need to detect the *direction of the gap* in the target and press the responding key as fast and accurately as possible. (**B**) Overview of all possible target locations. Values depicted next to the target represent the distance relative to the cue. Note, during the visual acuity task, the target locations are similar to those during the attention task, except this time there is no cue and no distractor

To characterize the size of the attentional spotlight, we used an adapted version of the cueing task that was developed by Robertson et al. (2013). Each trial started with the 450-ms presentation of a black circular fixation point as designed by Thaler et al. (2013), followed by a black dot that served as the cue (radius = 0.2°; 67 ms) on either the left or right side of the screen (Fig. 1A). Then, after a variable interstimulus interval (67, 135, or 210 ms), a target circle with a gap (black outer radius 0.5°, white inner radius 0.4°, gap width 0.15°; 67 ms) appeared either at the exact same location of the cue (hereafter referred to as “exact”), or at one of the three possible locations – “near,” “mid,” or “far” – in the same hemifield as the cue (valid trials). While the cue has some predictive qualities, it remains primarily exogenous in nature as it only indicates the general hemifield rather than the exact target location. Simultaneously with the target presentation, a circle of the same size but without a gap was presented 180° opposite the target as distractor stimulus. Stimulus locations, which could be above or below the horizontal meridian, were all positioned on an imaginary circle with a radius of 9° resulting in a total of 14 possible target locations (Fig. 1B). Participants were instructed to report as quickly and accurately via a keyboard response whether the target had a gap at the top (arrow up), bottom (arrow down), left (left arrow), or right (right arrow) of the circle. The cue signaled the display side of the upcoming target with 85.7 probability. On invalid trials, which were included to obtain an index of the cueing effect and to enforce fixation, targets appeared at the opposite side of the cue.

### Visual acuity task

To control for the possibility that our findings could be explained by visual acuity instead of covert attention, we included a visual acuity task at the start of the experiment. This task was identical to the attention task, except that there was neither a cue nor a distractor present. Whereas the “near,” “mid,” and “far” labels did not refer to the target-cue distance, as there was no cue and the targets were at equal peripheral distances from the fixation point, we maintained this terminology for consistency. We assessed both internal consistency and test-retest reliability of visual acuity measures across the 2-week period between sessions. This assessment served dual purposes: to establish whether attentional effects could be distinguished from basic visual acuity differences, and to validate the stability of perceptual measurements in our online experimental setup. Strong reliability would indicate consistent perceptual conditions across sessions, which is a prerequisite for interpreting attentional measures in this study design.

### Experimental design

The experiment started with the visual acuity block, wherein at random order targets appeared at each individual target location eight times resulting in 112 trials. The subsequent attention task consisted of three blocks, each containing 224 trials. Within each block, valid trials (n = 192) contained unique combinations of all experimental parameters: cue hemifield (left/right), target position along the horizontal meridian (above/below; except for exact trials), target distance (exact/near/mid/far), gap direction (top/bottom/left/right), and interstimulus interval (67/135/210 ms). By contrast, invalid trials (n = 32) contained eight observations per target location, with cue hemifield and interstimulus interval not being counterbalanced. Valid and invalid trials were randomly intermixed.

### Procedure

To prevent between-session variance associated with order, each participant first performed the visual acuity task, followed by the attention task in both Session 1 and Session 2. Prior to the experiment, we obtained informed consent, and participants were familiarized with the tasks through extensive written instructions and practice trials: five trials for visual acuity (no cue), five slow-motion trials with prolonged stimulus presentation (250 ms) for the attention task, and ten actual-speed trials for the attention task. Throughout all practice trials, participants received feedback. During the slow-motion trials, incorrect responses were followed by additional explanations to ensure task comprehension. No feedback was provided during the actual experiment. At the end of a block, accuracy and average reaction times were shown to keep participants motivated. This was followed by a timer of 30 s to force a break. However, participants were free to prolong their break.

To enforce fixation at trial onset, before each trial, a countdown (3 to 1 s) was incorporated at the center of the screen, after which a prompt indicated that participants had to press the space bar within 1,000 ms. If they pressed the space bar too late, they had to wait for 3,000 ms before the trial initiated.

### Questionnaires

At the end of Session 1, participants were asked for several demographic factors and continued with the questionnaires. As the results of the questionnaires are not discussed in this paper, a short description can be found in the Online Supplementary Materials .

## Results

### Preprocessing

Data were preprocessed in a Python (version 3.11) environment using custom scripts. Data of one participant were removed from both sessions due to chance level performance in Session 1 in the valid cue condition collapsed over target distances near, mid, and far. As the visual acuity block was designed to validate whether participants were able to perceive all targets, one participant was removed from the dataset due to below-level chance performance in the visual acuity block. Data were trimmed based on reaction times to exclude trials with premature responses (< 200 ms) or trials that indicated inattentiveness (> 2,000 ms). Consecutively, outliers were removed based on reaction times by using the mean plus 2.5 SD of the aggregated score within participants as a threshold. Data trimming and filtering resulted in 3.39% trial loss. The resulting dataset was used for error rate analysis. For the reaction time analysis, incorrect trials were then removed. This resulted in 30.66% overall trial loss for our reaction time analysis of Session 1. Data-cleaning procedures were repeated for the dataset of Session 2: 3.10% trial loss for the accuracy analysis and 27.23% overall trial loss after removing incorrect responses. See Online Supplementary Materials Fig. S1 for an extensive overview of the outlier removal procedure. The overall pattern of results did not qualitatively change after removing outliers. Statistical analyses were performed in JASP version 0.17.1.0. We have uploaded the experimental code, raw data, complete preprocessing script, and statistical results on the Open Science Framework (OSF) platform (https://osf.io/pa2y9/).

### Group level analyses

#### Visual acuity

**Fig. 2:**
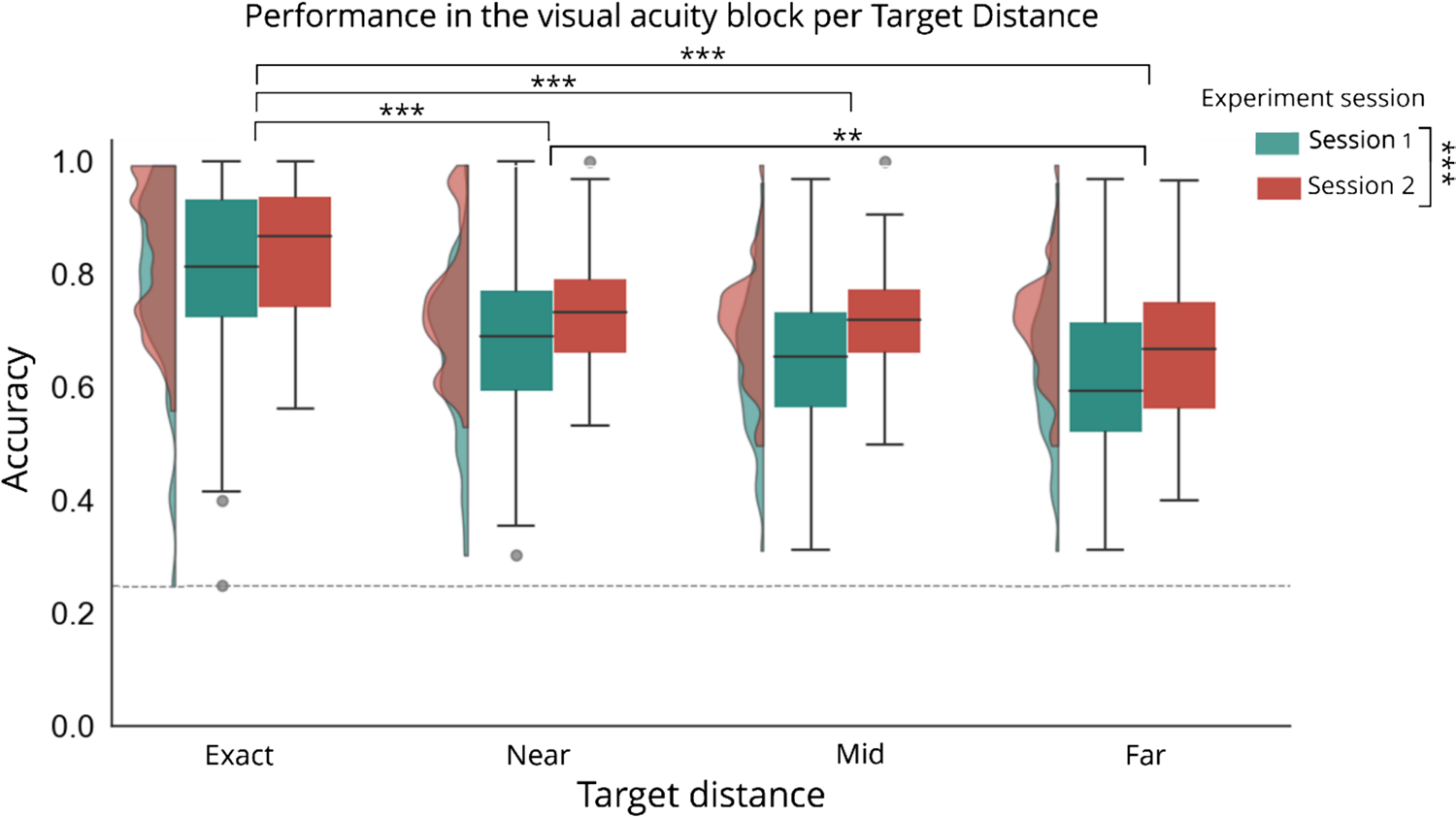
Performance (accuracy) in the visual acuity block per target location for Session 1 and Session 2. Dashed line indicates chance level. Performance was best at the exact target distance, and performance was poorer at the far compared to the near target distances. Performance was less accurate in Session 1 compared to Session 2. *p <.05, **p <.01, ***p <.001

We first investigated whether performance variation across target locations was due to differences in visual acuity rather than attentional zoom-lens effects induced by visual cues. To test this, we analyzed accuracy scores from the first experimental block, before the introduction of visual cues and distractors, using a repeated-measures ANOVA with within-subjects factors Session (Session 1 and Session 2) and Target Distance (exact, near, mid, far; locations coded by their distance from the future cue position). As visualized in Fig. 2, overal performance improved in Session 2 (main effect: *F*(1, 200) = 36.581, *p* <.001, *η*^*2*^_*p*_ =.155). Critically, although stimuli were presented equidistantly from fixation, a main effect of Distance (*F*(3, 200) = 21.645, *p* <.001, *η*^*2*^_*p*_ =.245) without an accompanying interaction (*F*(3, 200) =.594, *p* =.619) suggested performance declined with increasing distances from the horizontal meridian. While as expected performance was best for the targets presented closest to fixation (all *t*s > 4.664, all *p*s <.001), planned pairwise comparisons further revealed poorer performance at the far location compared to the near location (*t*(102) = −3.083, *p* =.007, Cohen’s *d* = −0.525), with reliably impaired performance at mid compared to near (*t*(102) = 1.124, *p* =.262) location and mid compared to far (*t*(102) = 1.959, *p* =.103) location. These baseline differences in visual acuity across display locations indicate that any subsequent attentional effects must be interpreted in light of these inherent performance variations.

#### Cueing effects

**Fig. 3:**
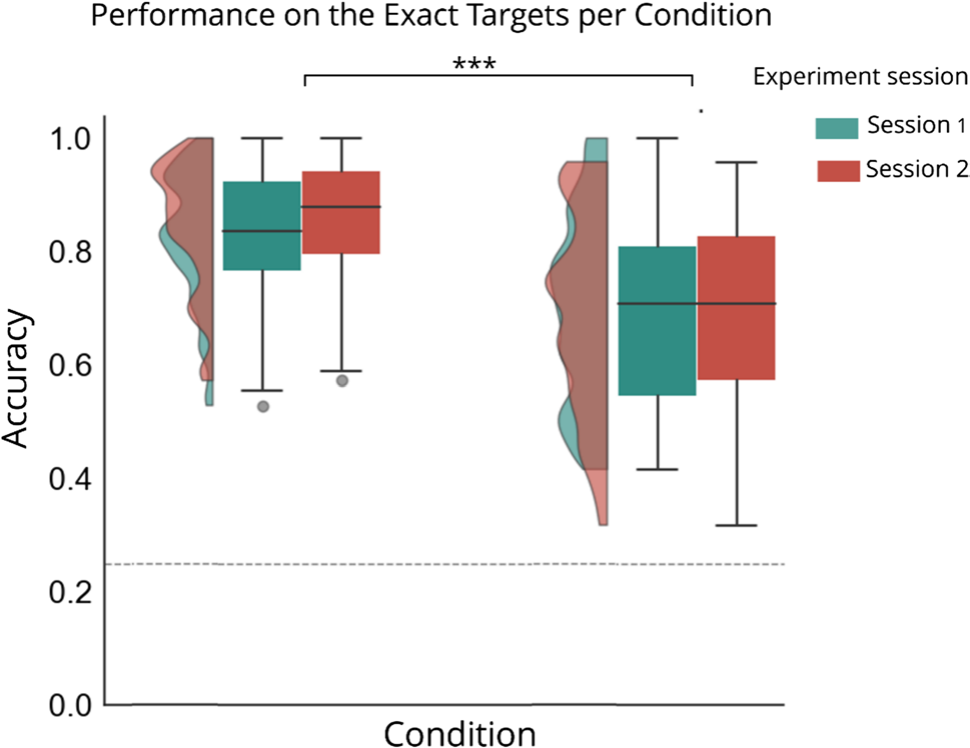
Performance (accuracy) for the exact trials in the different cue conditions (valid cues, invalid cues) for Session 1 and Session 2. Dashed line indicates chance level. Performance was better for the valid trials compared to the invalid trials. *p <.05, **p <.01, ***p <.001

Before characterizing details of the attentional spotlight, we first tested the ability of the cue to guide attention. For this purpose, we contrasted performance across cueing conditions using only exact target positions. As performance was not driven by interstimulus interval (*F*(2, 306) =.760, *p* =.468, *η*^*2*^_*p*_ =.001), we collapsed this factor over all trials in further analysis. As shown in Fig. 3, a repeated-measures ANOVA with within-subjects’ factors Cue condition (valid, invalid) and Session (1, 2) yielded a main effect of Cue condition (*F*(1, 100) = 32.072, *p* <.001, *η*^*2*^_*p*_ =.243), reflecting performance benefits at the cued location and costs at the non-cued location. There was no main effect of Session (*F*(1, 100) = 1.891, *p* =.172) nor an accompanying interaction (*F*(1, 100) =.390, *p* =.534). Together these results confirm previous findings in the literature that salient cues have the ability to summon attention.

#### Sharpness of the gradient

**Fig. 4:**
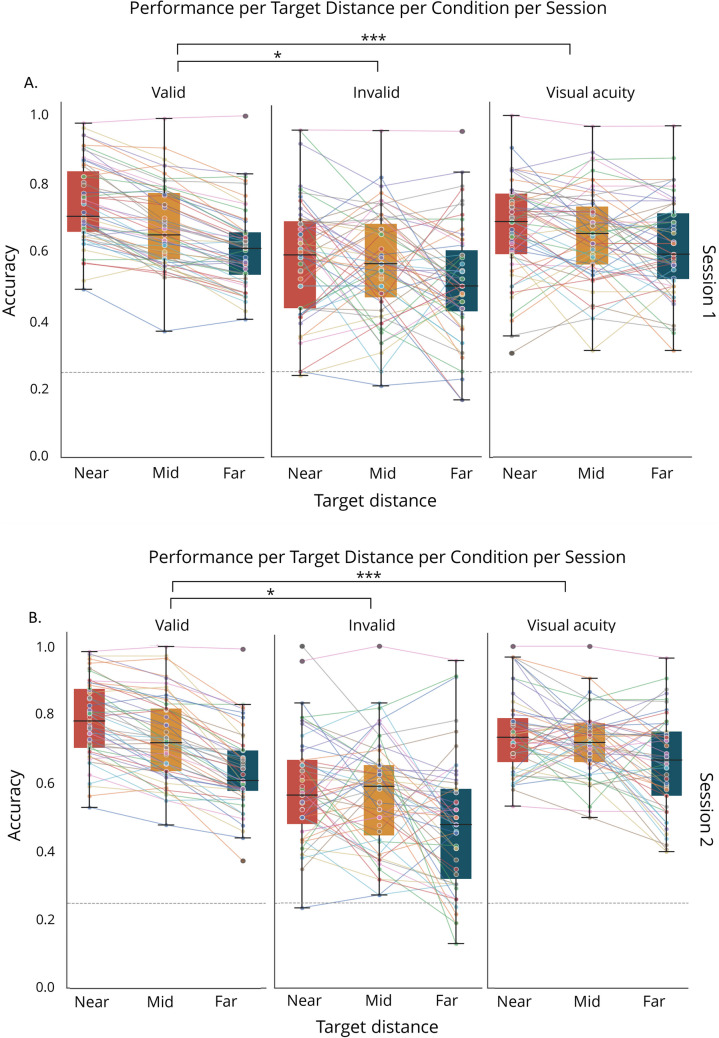
Representation of the performance slopes as a function of target distance per condition in (**A**) Session 1 and (**B**) Session 2. Connecting lines depict individuals. The dashed line indicates performance on chance level. *p <.05, **p <.01, ***p <.001

Having established reliable attentional guidance by our cue, we next set out to examine whether this exogenous cueing effect exhibited a spatial gradient. Whereas the previous analysis showed that the different target locations elicited differential effects on performance without guiding attention to a specific location, comparing performance slopes allowed us to investigate whether cueing modulates this sharpness gradient of the spotlight. Based on the trends in Fig. 4, we fitted linear models for individual accuracy data for condition and session. Target distance was coded ordinally (0 = near, 1 = mid, 2 = far), allowing us to quantify the change in performance per unit increase in distance.

Before comparing these individual slopes across conditions and session, we first assessed whether there was significant variation in intercepts and slopes across participants. To this end, all valid trials were analyzed with a generalized linear mixed model by using the glmer function in R. Factors included were target distance (centered at the near target distance) and session, along with their interaction and random slope and intercepts for individuals. A likelihood ratio test comparing this model to a reduced model without random slopes revealed that including random slopes significantly improved model fit (χ^2^(2) = 56.021, p < 0.001). This confirmed that participants varied significantly in how their accuracy changed with distance, validating our approach to examine individual differences in attentional gradients. Having established significant individual variation in attentional gradients, we proceeded with our planned analyses. A repeated-measures ANOVA with within-subjects’ factors Cue condition (valid, invalid, visual acuity (no cue)) and Session (Session 1 and Session 2) revealed a main effect of cue condition (*F*(2, 312) = 8.292, *p* <.001, *η*^*2*^_*p*_ =.050), with steeper performance gradients in the valid condition compared to both invalid (*t*(208) = 2.929, *p* =.004, Cohen’s *d* = 0.403) and visual acuity conditions (*t*(208)** =** −5.253, *p* <.001, Cohen’s *d* = 0.722) (Fig. 4). No significant gradient differences emerged between invalid and visual acuity trials (*t*(208) =.712, *p* =.477, Cohen’s *d* =.098). Neither the main effect of Session (*F*(1, 312) = 3.190, *p* =.075, *η*^*2*^_*p*_ =.010) nor the Session × Condition interaction reached significance (*F*(2, 312) =.084, *p* =.920, *η*^*2*^_*p*_ <.001). Critically, we also rigorously assessed potential practice effects within sessions by comparing the estimated slopes between blocks, using only data from the valid cue condition. A repeated-measures ANOVA with within-subjects’ factors Block (1, 2, 3) and Session (1, 2) revealed no significant main effects nor an interaction between block and session (all *F*s < 2.2, all *p*s >.15). This absence of systematic changes indicates that participants'attentional gradient patterns remained stable throughout the experiment, with no evidence of learning effects or strategic adaptations in how they utilized the spatial cues. This stability is consistent with the rapid, automatic deployment characteristic of exogenous attention mechanisms and suggests that the attentional gradients measured in our paradigm reflect fundamental properties of spatial attention rather than task familiarity or practice effects (Carrasco, 2011; Müller & Rabbitt, 1989).These results demonstrate that while target distance affects performance across all conditions, this effect is significantly more pronounced when attention is directed to the cued location, potentially providing a stable index of the attentional gradient of the spotlight. The stability of these attentional gradient patterns across sessions at the group level provided a foundation for subsequent individual-level analyses.

#### Reaction times

To elucidate potential differences in reaction times based on target location, we repeated our statistical procedures as described above focusing exclusively on correct trials. Notably, unlike the accuracy data, the visual acuity condition showed no effect of Distance on reaction times. When comparing the reaction times on the exact locations, results revealed faster reaction times for the valid condition compared to the invalid condition. Slope analysis indicated a steeper increase in reaction times as a function of target distance in the valid condition compared to both the invalid and the visual acuity condition. As reaction time is not our primary outcome measure, detailed statistical findings are reported in the Online Supplementary Materials .

### Individual level analysis

#### Between-subjects variance in accuracy, reaction time, and slopes

**Fig. 5:**
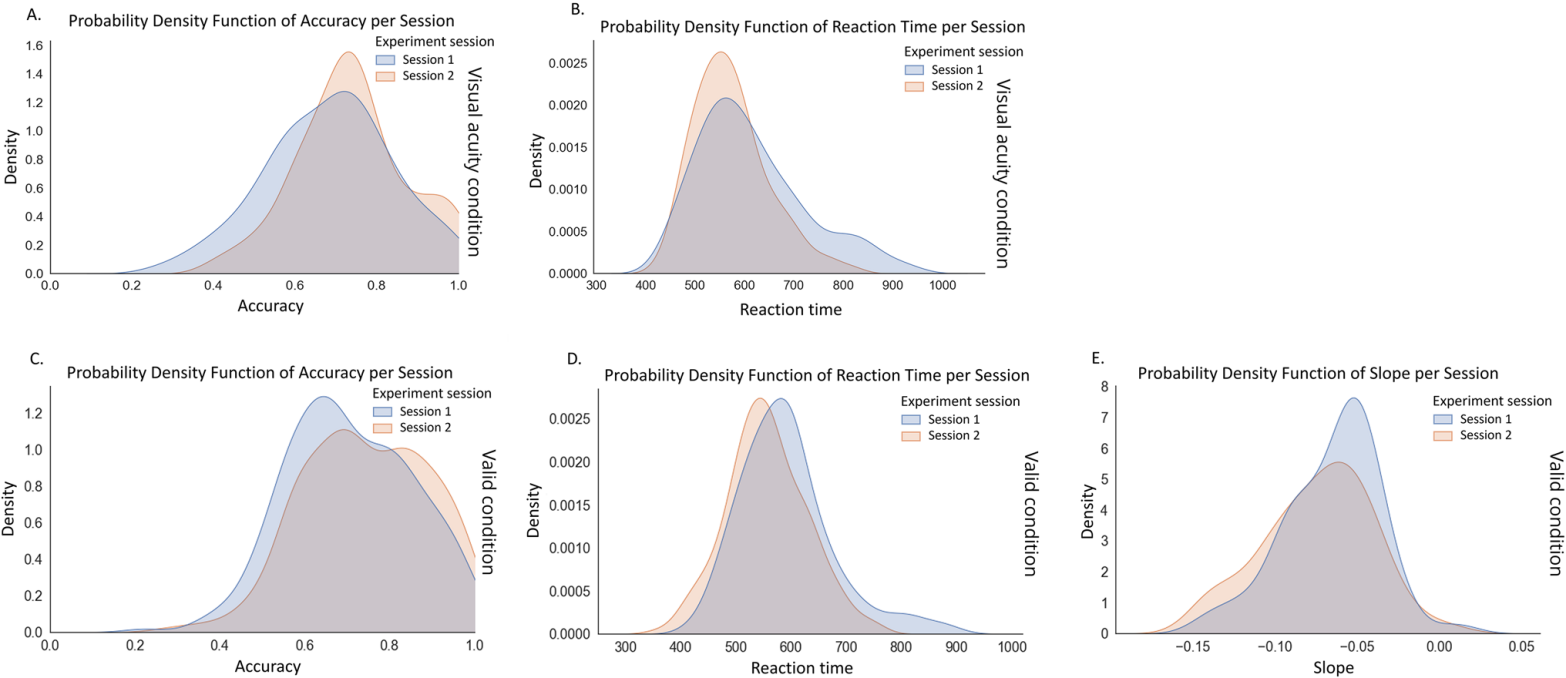
Probability functions representing kernel density estimates for (**A**) accuracy in the visual acuity condition, (**B**) reaction time in the visual acuity condition, (**C**) accuracy in the valid condition, (**D**) reaction time in the valid condition, and (**E**) slope fitting in the valid condition. Data are split between the two sessions, with blue representing Session 1 (test) and red representing Session 2 (retest)

With meeting the prerequisite of the stability of our findings over time on a group level, we proceeded to investigate the potential of our task to infer individual differences. First, Fig. 5 shows the distribution of our data for Session 1 and Session 2 for the accuracy, reaction times, and performance decline slopes for the visual acuity condition and the valid condition. The distributions suggest intra-individual variability regarding accuracy (Figs. 5A, C), reaction time in the visual acuity condition (Fig. 5B), and slope fitting data (Fig. 5E). For reaction time (Fig. 5D), we see little variability, and the small shift to the left for the distribution in Session 2 compared to that in Session 1 implies that learning effects are present. The general overlap between all density plots hints towards similarity of the data in Session 1 and Session 2.

#### Internal consistency

To asesss the trait-or-state like characteristics of the attentional spotlight, we first examined whether our cueing paradigm yielded high internal consistency within individual sessions Using permutation-based split-half reliability, we computed 5,000 random splits of all trials (clustered to mitigate trial sampling effects; Parsons et al., 2019; Pronk et al., 2022), and derived Spearman-Brown-corrected reliability estimates for both accuracy and reaction times across all task conditions (visual acuity, valid, invalid).

For the visual acuity condition, reliability estimates for accuracy and reaction time were robust. In Session 1, accuracy reliability reached r =.818, 95% confidence interval *(CI)* = [.735,.882], while reaction time reliability was r =.976, *CI* = [.964,.985]. Similarly, in Session 2, accuracy reliability was *r* =.772, *CI* = [.674,.852], with reaction time reliability at r =.968, CI = [.951,.981]. These results indicate stable perceptual baseline performance within each session. We further calculated internal consistency separately for each target distance (Table 1); the somewhat lower reliability in these sub-conditions is likely due to reduced trial numbers. The valid cue condition demonstrated even stronger internal consistency. Session 1 yielded accuracy reliability of r =.962, CI = [946,.975] and reaction time reliability of r =.989, CI = [.981,.994]. Session 2 showed comparable values with accuracy reliability at r =.970, CI = [.955,.981] and reaction time reliability at r =.988, CI = [.980,.993]. For the invalid condition, we observed identical estimates to the valid condition for, respectively, accuracy and reaction time: Session 1: r =.962, CI = [946,.975]; Session 2: r =.970, CI = [.955,.981]; Reaction time Session 1: (r =.989, CI = [.981,.994]; Session 2: r =.988, CI = [.980,.993].

**Table 1:** Results of the split-half analyses of the reaction times and accuracy with 5,000 permutations

		Reaction times		Accuracy	
Condition	Target distance	Session 1	Session 2	Session 1	Session 2
Visual acuity	Clustered	.976, CI [.964,.985]	.968, CI [.951,.981]	.818, CI [.735,.882]	.772, CI [.674,.853]
	Exact	.876, CI [.829,.919]	.833, CI [.758,.898]	.535, CI [.341,.694]	.594, CI [.433,.727]
	Near	.910, CI [.868,.944]	.887, CI [.830,.932]	.593, CI [.422,.730]	.547, CI [.351,.701]
	Mid	.936, CI [.906,.961]	.906,CI [.861,.943]	.563, CI [.386,.710]	.212, CI [-.104,.457]
	Far	.920, CI [.881,.952]	.888, CI [.837,.929]	.587, CI [.416,.729]	.665, CI [.510,.790]
Valid	Clustered	.989, CI [.981,.994]	.988, CI [.980,.993]	.962, CI [.946,.975]	.970, CI [.955,.981]
	Exact	.973, CI [.958,.984]	.971, CI [.956,.982]	.928, CI [.893,.954]	.933, CI [.900,.960]
	Near	.967, CI [.950,.980]	.969, CI [.953,.982]	.890, CI [.846,.927]	.919, CI [.880,.951]
	Mid	.962, CI [.941,.979]	.961, CI [.941,.978]	.869, CI [.815,.914]	.907, CI [.863,.943]
	Far	.958, CI [.938,.975]	.953, CI [.934,.970]	.840, CI [.769,.898]	.840, CI [.770,.897]
Invalid	Clustered	.934, CI [.902,.960]	.930, CI [.893,.960]	.819, CI [.736,.884]	.852, CI [.782,.907]
	Exact	.805, CI [.727,.875]	.769, CI [.648,.858]	.727, CI [.596,.825]	.740, CI [.610,.837]
	Near	.837, CI [.765,.898]	.757, CI [.635,.850]	.616, CI [.457,.743]	.536, CI [.319,.702]
	Mid	.747, CI [.650,.829]	.779, CI [.687,.855]	.565, CI [.364,.719]	.587, CI [.401,.735]
	Far	.768, CI [.650,.829]	.694, CI [.660,.856]	.575, CI [.398,.717]	.685, CI [.540,.798]

Spearman-Brown-corrected r-coefficients are reported with 95% confidence intervals of the bootstrapped distribution of estimates in square brackets

#### Slope reliability

While split-half reliability is well suited for assessing the consistency of aggregated performance measures like accuracy and reaction times, slope estimates derived from condition-wise differences (e.g., attentional gradients across target distances) are more sensitive to trial sampling variability.^1^ As a result, we employed a bootstrapping approach for slope estimation, which allowed us to generate robust confidence intervals around individual slopes by resampling the underlying trial data. This approach accounts for the intrinsic uncertainty in difference-based metrics and provides a more nuanced estimate of reliability for within-subject attentional gradients. After establishing high internal consistency for task performance measures, we evaluated the reliability of our key metric of interest: the attentional spotlight gradient, operationalized as the slope across target distances. Given the sensitivity of slope estimates to trial sampling, we employed a bootstrapping approach, resampling trials with replacement (5,000 iterations per participant) to derive confidence intervals for each individual's slope. This method revealed that 98% of participants demonstrated reliably negative slopes (i.e., 95% CI (Bayesian) upper bound < 0). When split by session, results were nearly identical: during the first session 98.2% of participants showed reliably negative slopes with a group-level CI [−0.0660, −0.0645], while at retest 98.0% of participants maintained this pattern with a group-level CI [−0.0748, −0.0734]. These results confirm that although slope steepness may vary across trial samples, the direction of the attentional gradient remains negative and consistently measurable within participants.

1. When assessing the internal consistency of the slopes, our first method was to conduct a split-half bootstrapping approach with 5,000 iterations – designed to balance conditions. This resulted in the surprising finding of consistently negative correlations between the two halves of slope estimates within participants. These negative correlations likely reflect restricted between-subject variance in slope estimates – most participants exhibited similarly negative slopes, leaving minimal interindividual variability to support stable rank-order differences. Random trial-level fluctuations, combined with the constraint of our balancing procedures, may have introduced systematic differences between the split-half analyses, resulting in negative rather than near-zero correlations typically expected from restricted variance alone.

#### Test-retest reliability

**Fig. 6:**
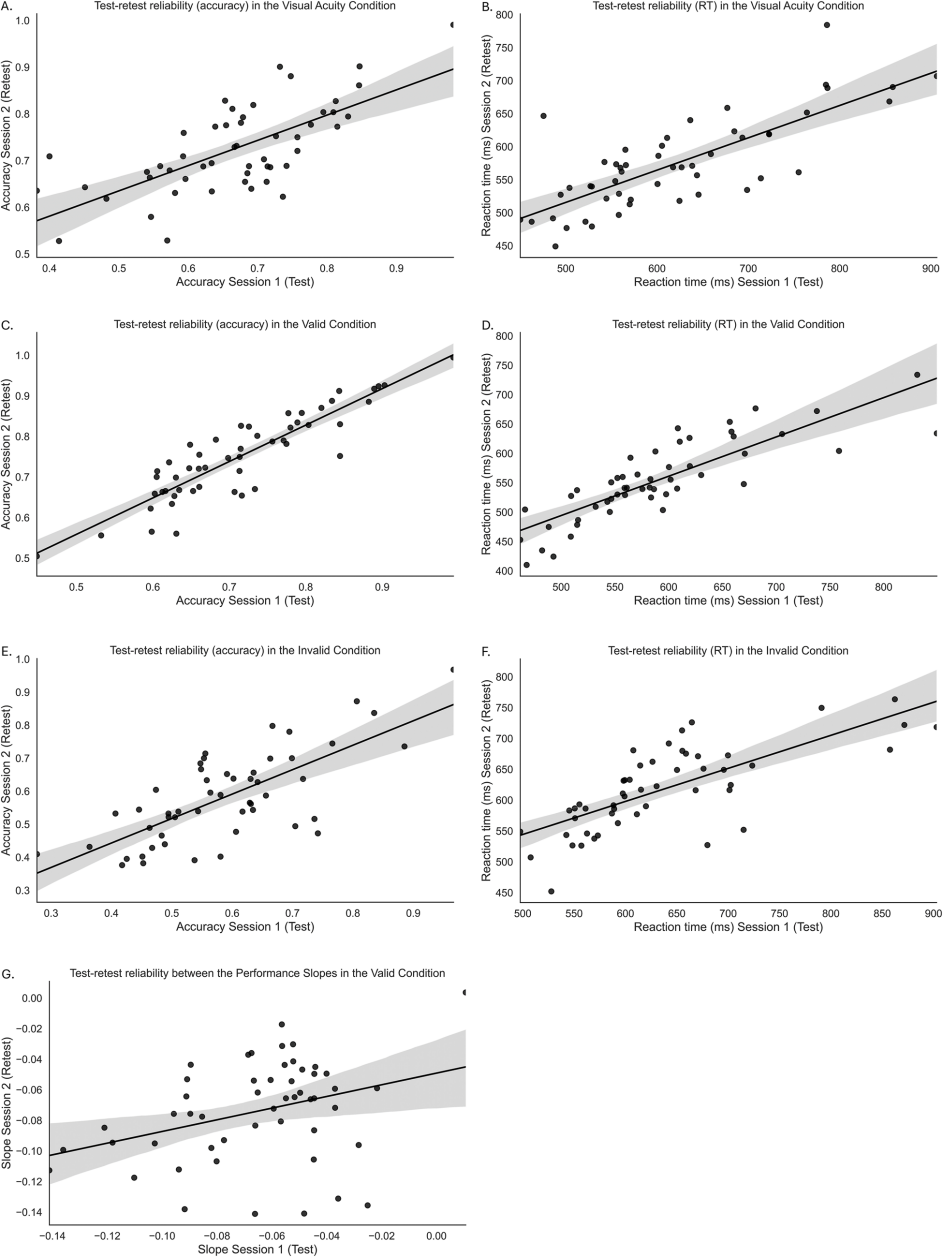
Test-retest analysis of accuracy (left panels) and reaction time (right panels) for Session 1 (x-axis) and Session 2 (y-axis) in the (**A, B**) visual acuity condition, (**C, D**) valid condition, and (**E, F**) invalid condition. Panel **G** shows the test-retest reliability of the slopes between both sessions in the valid condition. The shaded area around the regression line represents the 95% confidence interval

Having established both the internal reliability of the attentional gradients (slopes) within participants and the presence of significant individual variation in these patterns, we then confirmed the stability of test-retest reliability for individual performance in terms of accuracy and reaction times across all conditions (all *r*s >.6, Fig. 6A-F, Table 2). With this foundation, we proceeded to assess the 2-week test-retest reliability of the individual performance gradients (slopes) (Fig. 6G). The Spearman correlations between the performance slopes in Session 1 and Session 2 for the valid condition was weak: valid (*r* =.273, *CI* [-.001,.559]), suggesting low stability in individual attentional gradient patterns over time (Fig. 6G). For completion, we calculated the test-retest reliability of the slopes based on reaction times, which yielded similar results for the valid condition (*r* =.333, *CI* [-.063,.557]). These findings limit the paradigm’s suitability for research on individual trait differences in the size of the attentional spotlight gradient.

**Table 2:** Results of the test-retest reliability between Session 1 and Session 2 for reaction times and accuracy in both the valid condition and the visual acuity condition

		Reaction time		Accuracy	
Condition	Target distance	Spearman r	95% CI	Spearman r	95% CI
Visual acuity	Clustered	.717	[.434, 1.00]	.633	[.350,.916]
	Exact	.665	[.382,.948]	.456	[.173,.739]
	Near	.700	[.417,.983]	.536	[.253,.820]
	Mid	.701	[.418,.984]	.392	[.109,.675]
	Far	.667	[.384,.950]	.601	[.318,.884]
Valid	Clustered	.861	[.578, 1.00]	.875	[.592, 1.00]
	Exact	.841	[.558, 1.00]	.824	[.541, 1.00]
	Near	.813	[.531, 1.00]	.743	[.460, 1.00]
	Mid	.844	[.561, 1.00]	.819	[.536, 1.00]
	Far	.845	[.562, 1.00]	.832	[.549, 1.00]
Invalid	Clustered	.739	[.456, 1.00]	.660	[.377,.943]
	Exact	.706	[.423,.989]	.584	[.301,.867]
	Near	.581	[.298,.864]	.541	[.258,.824]
	Mid	.561	[.278,.844]	.418	[.135,.701]
	Far	.608	[.325, 1.00]	.349	[.066,.632]

Spearman-Brown r-coefficients are reported with 95% confidence interval estimates in square brackets

## Discussion

While the flexible nature of the attentional spotlight is well documented (e.g., Belopolsky & Theeuwes, 2010; Castiello & Umiltà, 1990; Kolnes et al., 2024), the existence of stable individual differences in spotlight characteristics remains poorly understood. This study therefore investigated whether, in addition to state-dependent variations, the attentional spotlight also entails trait-like individual differences. Using a spatial cueing paradigm, we mapped the gradient of attention by presenting targets at varying distances from validly or invalidly cued locations. At the group level, our results revealed a clear attentional gradient: performance declined systematically as the distance between cue and target increased. However, despite these robust group-level effects, individual differences in the spotlight gradient proved unstable across a 2-week interval, suggesting that either trait-like variations in attentional breadth may be difficult to capture with the current paradigm, or that such individual differences are not as stable as previously thought.

Previous research investigating differences in modulations of the size of the attentional spotlight has primarily relied on tasks like the Navon paradigm or hierarchical shape tasks (Colzato et al., 2010; Kimchi & Palmer, 1982; McKone et al., 2010; Navon, 1977). However, these measures are confounded by various cognitive processes, including executive functioning and cognitive control, making it challenging to isolate the specific element of the attentional spotlight (Busch et al., 2024; Dale & Arnell, 2013; Medaglia et al., 2017). To address these limitations, we adapted the cueing paradigm of Robertson et al. (2013), which offers two key advantages: it provides a relatively pure measure of spotlight size and yields a continuous measure of the attentional gradient.

The current study expanded upon the paradigm of Robertson et al. (2013) in two important ways. First, we quantified the attentional gradient by fitting linear slopes to performance decline as a function of target distance. This approach provided a reliable estimate of gradient sharpness, as evidenced by the clear linear trends in our data. Importantly, our bootstrap analyses demonstrated that these slopes were reliably negative within a single experimental session, with over 98% of participants showing a consistent negative gradient pattern in both test and retest sessions. While this consistency in direction is noteworthy, our analyses focused primarily on establishing the presence of negative gradients rather than confirming within-session reliability of gradient steepness itself. This distinction is important for interpreting individual differences in attentional gradient characteristics. Some might argue these gradients simply reflect attentional shifts proportional to cue-target distance (e.g., Heitz & Engle, 2007; Shulman et al., 1979). However, such interpretations typically emerge from paradigms employing much longer interstimulus intervals (500–4,500 ms) that allow time for attentional repositioning, making this interpretation in our current paradigm untenable due to the substantially shorter interstimulus intervals. The temporal constraints in our design argue against shift-based interpretations as viable explanations. Second, we systematically mapped visual acuity across all target locations by assessing how well participants could identify the target gaps without cues or distractors being present. This condition was administered to rule out the possibility that differences between participants in cueing effects could be explained by differences in visual acuity along the vertical plane. In other words, were participants able to perceive the target gaps at all? Indeed, participants performed above chance level, confirming that they were able to perceive the directions of the target gaps. We attribute the inherent variations in accuracy – but not in reaction times – across display conditions to previously reported visual acuity heterogeneity across the visual field, such as performance decrease for oblique compared to cardinal directions and horizontal-vertical anisotropy (Barbot et al., 2021; Carrasco et al., 2001). Targets were only briefly presented in an uncued location, necessitating sustained attention across the periphery. The role of attention in the “visual acuity” task might also explain why the test-retest reliability of performance here was lower than would be expected if pure visual acuity had been measured. Despite this limitation, however, the visual acuity mapping approach allowed us to disentangle attentional effects, as measured via our slope-fitting procedure, from baseline perceptual differences. Future studies could expand the number of cue and target locations, allowing for more extensive mapping of the attentional field whilst simultaneously reducing cue predictability.

Importantly, our successful replication of both standard exogenous cueing effects (Posner, 1980) and Robertson et al.’s (2013) attentional gradient in an online environment validates this paradigm for large-scale web-based studies. This finding aligns with growing evidence supporting the reliability of online experiments (e.g., Bazilinskyy & Winter, 2018; Germine et al., 2012). While our results suggest limitations in measuring individual differences, the paradigm's advantages for group-level comparisons opens new avenues for research. Future studies could employ this more precise measure of attentional gradients to reassess previously reported group differences in local and global processing (Colzato et al., 2010; Ten Brink et al., 2024; McKone et al., 2010; Colzato, 2010), whether between clinical populations, as in Robertson et al.'s (2013) original work, which compared individuals with ASD to neurotypicals, cultural groups, or other demographic factors.

Despite demonstrating robust group-level effects that validate the cueing paradigm’s utility and stable general performance as indicated by the test-retest reliability of accuracy and reaction time, our investigation into individual differences in the attentional gradient yielded unreliable effects. The spatial gradient of attention, as measured by our slope indices, showed low test-retest reliability, suggesting an absence of stable individual differences in a 2-week time interval. Importantly, the absence of learning effects and the stability of our basic performance metrics (i.e., accuracy and reaction times), the absence of the main effect of interstimulus interval, and the negative attentional gradient within sessions indicate an absence of systematic eye movements (though direct oculomotor monitoring was not implemented) and no strategic adaptations in how participants utilized the spatial cue.

This dissociation in our results raises important questions about the stability of attentional spotlight characteristics over time. Despite clear evidence that our measurements themselves were reliable (supporting the validity of online testing environments) and that our generalized linear mixed model approach demonstrated significant individual variation in slopes, we found no evidence that this attentional gradient metric was stable between sessions. This pattern suggests two possible interpretations. While one possibility is attentional gradient characteristics primarily reflect state-dependent variations rather than stable traits, we believe it is more likely that our specific cueing paradigm enforced a relatively constrained shape of the attentional gradient across participants. As Hedge et al. (2018) argue, well-established experimental effects often show limited between-subject variability, making them less suitable for detecting individual differences. This interpretation is further supported by the surprising finding of consistently negative correlations between randomly split slope estimates within a single session. Rather than indicating unreliability per se, these negative values likely reflect a floor effect in variability – where all participants show similarly negative slopes, leaving minimal room for stable rank-order differences to emerge. In such cases, even small trial-sampling fluctuations can reverse the relative ranking of participants, thus producing negative correlations. Accordingly, our bootstrapping analysis confirmed that slope estimates were consistently negative and statistically reliable within individuals, suggesting that the metric is internally stable but may lack sufficient between-subject variance to support cross-session correlations. Additionally, the sensitivity of our measurement approach may have played a crucial role. While the paradigm effectively captured overall attentional effects, specific task parameters might have constrained our ability to detect subtle individual spotlight characteristics in the neurotypical population. For example, the size and location (either left or right) of our attentional cue might have imposed constraints on the natural spread of attention, leading to a stimulus-driven attentional gradient (Taylor et al., 2015). If the influence of cue size on the spotlight exceeded individuals’ biases in attentional breadth, our paradigm might have masked underlying trait differences.

Regarding the applied methodology in the current experiment, it is important to note that the cue had some predictive value of the upcoming target as it indicated the hemifield of the target with a high probability, although it did not signal the precise target location. Given this imprecise spatial nature of the cue and the short interstimulus intervals within the exogenous timeframe, we believe our measurements predominantly captured exogenous attention processes, although we cannot conclusively rule out endogenous influences. Therefore, to decrease the potential incentive to search for the cue to decrease the difficulty of suppressing the distractor which shares task-relevant features (Gibson & Kelsey, 1998; Weichselbaum et al., 2018; Weichselbaum & Ansorge, 2018), future studies could expand the number of cue locations (simultaneously allowing for more extensive mapping of the attentional field as well) and make the cue fully non-predictive of the upcoming target location.

These methodological considerations can be further contextualized by comparing our findings to the study of Robertson et al. (2013), in which clear spotlight differences between neurotypical individuals and those with ASD were found. This comparison suggests that while spotlight characteristics may vary meaningfully between distinct populations, the variation within our neurotypical sample might have been too subtle to reliably detect. In other words, while the current paradigm may not be sensitive enough to capture individual differences within a neurotypical population, it can still effectively distinguish between-group differences in attentional processing. Future studies could investigate these possibilities by systematically varying cue sizes and examining the paradigm's sensitivity across different populations.

In conclusion, our study demonstrates that while the cueing paradigm robustly captures the spatial distribution of attention at the group level, individual differences in spotlight characteristics showed limited stability over a 2-week period. This finding highlights an important distinction between group-level effects and individual differences in attention research. Although the homogeneity of our neurotypical sample and inherent task characteristics may have limited our ability to detect subtle trait differences in the spotlight gradient, the paradigm offers distinct advantages over traditional measures like Navon tasks. Its relative purity as a measure of attentional processing, combined with its demonstrated viability in online testing environments, makes it particularly valuable for investigating between-group differences in attentional spotlight characteristics.

## Supplementary Information

Below is the link to the electronic supplementary material.

Supplementary file1 (DOCX 69 KB)

## Data Availability

Data and analyses scripts to reproduce the results are available via the Open Science Framework: https://osf.io/pa2y9/

## Code availability

Code is available via the Open Science Framework: https://osf.io/pa2y9/
