# Supplementary material for: The applicability of a cueing paradigm to study individual differences in the spotlight of attention
Source: Atten Percept Psychophys. 2025 Jul 29;87(8):2250–64. doi: 10.3758/s13414-025-03136-0 (PMC12568912; doi:10.3758/s13414-025-03136-0)
Supplement: Supplementary file 1 — Supplementary file1 (DOCX 69 KB) [file 13414_2025_3136_MOESM1_ESM.docx]

**Supplementary Materials**

*Broad Autism Phenotype Questionnaire (BAPQ).* The BAPQ is a 36-item questionnaire developed to measure autistic traits in the general population (Hurley et al., 2007). It identifies individuals with broader autism phenotype (BAP) which refers to the characteristics of autism but to a milder extent. The 3 subscales of the questionnaire (aloof personality, rigid personality and pragmatic language) parallel deficits in social communication, stereotyped-repetitive behaviors and social language deficits which are the key components of autism (American Psychiatric Association, 2013). Participants are asked to rate how frequently each statement applies to them on a 6-point Likert-scale, ranging from very rarely to very often. The overall BAP characteristics and subscale are calculated by averaging the corresponding items. Higher scores indicate higher levels of phenotypic expression.

*Self-report psychopathy (SRP).* The SRP is a self-report measure of psychopathy in the subclinical population (Massa & Eckhardt, 2017; William et al., 2003). Psychopathy is a personality construct marked by interpersonal facets (e.g., manipulation), affect (e.g., cold affect, lack of empathy), lifestyle (e.g., thrill-seeking behavior) and antisocial behaviors (e.g., criminal offences) (Neumann et al., 2015). The short form of the questionnaire consists of 29 items in which participants rate the degree to which they agree with the statements on a 5-point scale from *Disagree strongly* to *Agree strongly*. The four subscales provide insights in which facet of psychopathy is most prominently present and the total score reflects a valid measure of psychopathy in forensic and non-forensic populations.

*Strength Difficulties Questionnaire (SDQ).* The 25-items in the SDQ comprise 5 scales with 5 items each, indicating Emotional problems, Conduct problems, Hyperactivity, Peer problems and Prosocial behavior (sdqinfo.org). Participants rate each question on a 3-point scale from *Not true*, to *Somewhat True* to *Certainly True*. Scores within a subscale range from 0 to 10, with higher scores indicating higher experienced difficulties. A Total Difficulties Score can be generated by summing the first 4 subscales (all except the prosocial scale). Follow-up question are developed to enquire information about the impact of the severity in terms of everyday life interferences, e.g., chronicity, distress, social impairment and burden to others.

**
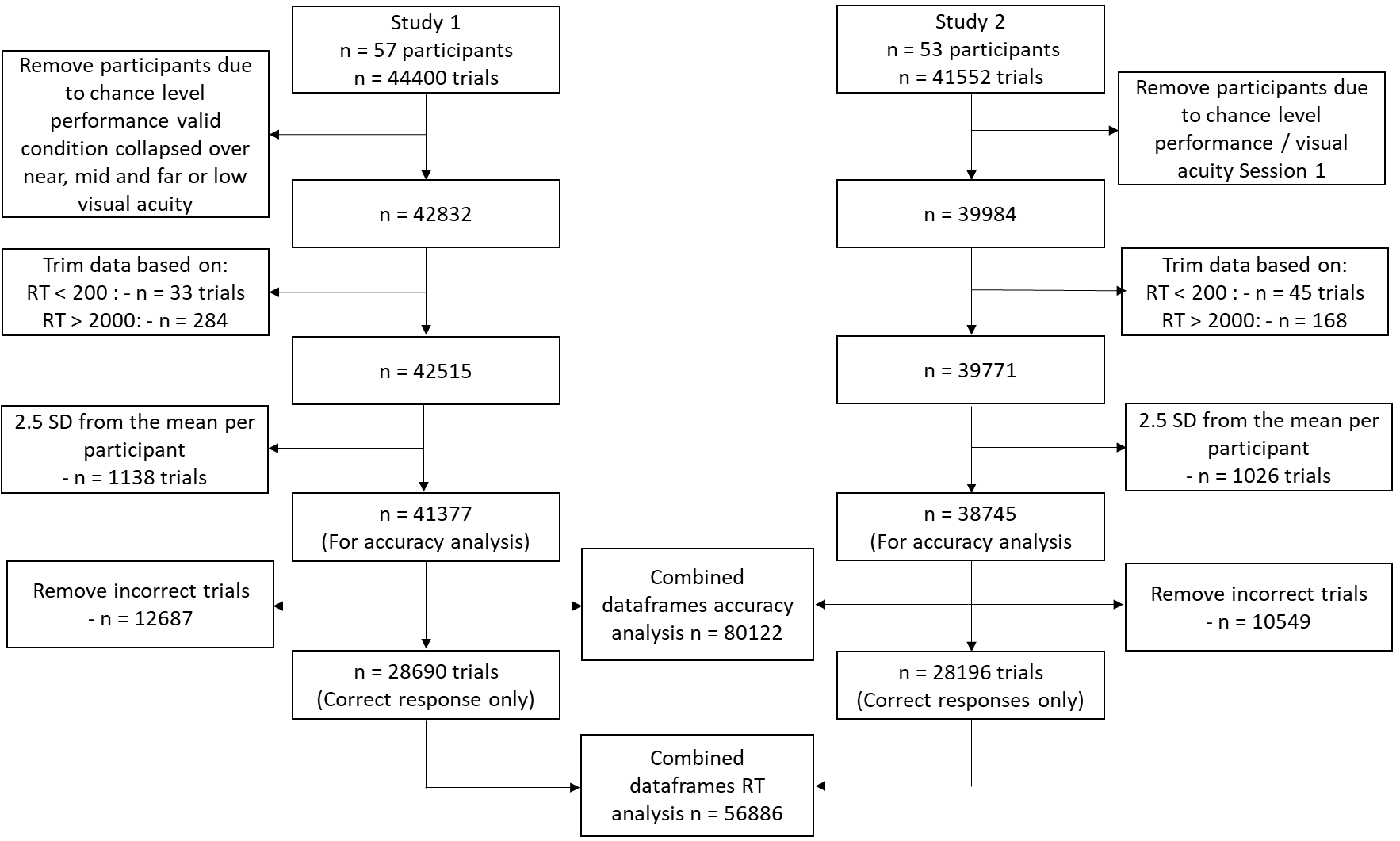
**

Figure S1. Outlier-removal overview.

**Reaction time analysis**

*Visual acuity.* We first investigated whether the different display locations elicited variations in reaction time, by analyzing reaction time from the first experimental block, before the introduction of visual cues and distractors. The results of the repeated measures ANOVA with within-subjects factors Session (Session 1 and Session 2) and Distance (exact, near, mid, far; locations coded by their distance from the future cue position) indicated faster overall reaction times in Session 2 (main effect: *F*(1, 200) = 67.411, *p* < .001, *η^2^_p_* = .252). There was no effect of Distance (*F*(3, 200) = 2.094, *p* = .102, *η^2^_p_* = .030) nor an interaction between Distance and Session (*F*(3, 200) = 0.580, *p* = .629, η^2^_p_ = .009).

*Cueing effects.* When comparing the reaction times on the exact locations between the valid and invalid trials, we again find a main effect of Session, indicating faster RTs in Session 2 (*F*(1, 100) = 12.913, *p* < .001, *η^2^_p_* = .114). The result yielded faster RTs for the valid condition compared to the invalid condition (*F*(1, 100) = 22.839, *p* < .001, η^2^_p_ = .186), with a significant Session × Condition interaction (*F*(1, 100) = 8.268, *p* = .005, *η^2^_p_ =* .076).

*Sharpness of the gradient.* Slopes for the reaction time analysis were calculated using the same approach as previously for performance. In short, target distance was coded ordinally (0 = near, 1 = mid, 2 = far), allowing us to quantify the change in RT per unit increase in distance. In line with the slopes based on accuracy, a repeated measures ANOVA with within subjects’ factors Cue condition (valid, invalid, visual acuity) and Session (1, 2) revealed a main effect of cue condition (*F*(2, 150) = 6.070, *p* = .003, *η^2^_p_* = .075) meaning significantly longer reaction time as a function of increasing target distance in the valid condition compared to the invalid condition (*t*(103) = 3.255, *p* = .004, Cohen’s *d* = -0.443) and compared to the visual acuity condition (*t*(103) **=** 2.704, *p* = .021, Cohen’s *d* = .368). Our results yielded no slope difference between visual acuity and invalid (*t*(103) = .552, *p* = .846, Cohen’s *d* = .075). Slopes were steeper in Session 2 compared to Session 1 (*F*(1, 150) = 4.193, *p* = .042, *η^2^_p_* = .027) with no accompanying Session x Condition interaction (*F*(2, 150) = .488, *p* = .615, *η^2^_p_* = .006).
